# Sterilization and sanitizing of 3D-printed personal protective equipment using polypropylene and a Single Wall design

**DOI:** 10.1186/s41205-021-00106-8

**Published:** 2021-06-11

**Authors:** Karstan Luchini, Shelly N. B. Sloan, Ryan Mauro, Aspram Sargsyan, Aundrea Newman, Purnadeo Persaud, Daniel Hawkins, Dennis Wolff, Jeff Staudinger, Bradley A. Creamer

**Affiliations:** 1 Department of Basic Sciences, Kansas City University, College of Medicine, Farber-McIntire Campus, Joplin, MO USA; 2 Department of Biology and Environmental Health, Missouri Southern State University, Joplin, MO USA; 3 Lead Systems 3D Engineer- Nemotech LLC, Joplin, MO USA

**Keywords:** Personal protective equipment (PPE), COVID-19, Reusability, Sterilize, Polypropylene (PP), Polylactic acid (PLA), Thermoplastic polyurethane (TPU), Biomedical waste (BMW), Fused filament fabrication (FFF)

## Abstract

**Background:**

The emergence of the severe acute respiratory syndrome coronavirus 2 (SARS-CoV-2) pandemic during the fall of 2019 and into the spring of 2020 has led to an increased demand of disposable N95 respirators and other types of personal protective equipment (PPE) as a way to prevent virus spread and help ensure the safety of healthcare workers. The sudden demand led to rapid modification, development, and dissemination of 3D printed PPE. The goal of this study was to determine the inherent sterility and re-sterilizing ability of 3D printed PPE in order to provide sterile equipment to the healthcare field and the general public.

**Methods:**

Samples of polylactic acid (PLA), thermoplastic polyurethane (TPU) (infill-based designs) and polypropylene (single-wall hollow design) were 3D printed. Samples were inoculated with *E. coli* for 24 h and then sanitized using various chemical solutions or heat-based methods. The samples were then incubated for 24- or 72-h in sterile LB medium at 37°C, and bacterial growth was measured by optical density at 600nm. Statistical analysis was conducted using GraphPad Prism v8.2.1.

**Results:**

Significant bacterial growth was observed in all PLA and TPU based samples following re-sterilization, regardless of the methods used when compared to controls (*p* < 0.05). The single-walled hollow polypropylene design was not only sterile following printing, but was also able to undergo re-sanitization following bacterial inoculation, with no significant bacterial growth (*p* > 0.05) observed regardless of sanitization method used.

**Conclusion:**

The cost effectiveness, ease of sanitization, and reusability of 3D printed PPE, using our novel single-walled polypropylene design can help meet increased demands of PPE for healthcare workers and the general public that are needed to help decrease the viral transmission of the coronavirus disease of 2019 (COVID-19) pandemic. 3D printing also has the potential to lead to the creation and production of other sterile material items for the healthcare industry in the future. The ability to re-sterilize 3D printed PPE, as our design shows, would also contribute less to the increase in biomedical waste (BMW) being experienced by COVID-19.

**Supplementary Information:**

The online version contains supplementary material available at 10.1186/s41205-021-00106-8.

## Background

The emergence of the COVID-19 pandemic during the fall of 2019 and into the spring of 2020 has led to a heightened demand of disposable PPE, including N95 respirator masks and single use disposable facemasks, as a way to prevent virus spread and help ensure the safety of healthcare workers and others who may come in contact with the virus. As it has become clear that the primary mode of transmission of coronavirus is through respiratory droplets, the use of facemasks in the healthcare setting has shown to limit the transmission of infectious agents [1–4]. In addition, elevated recommendations at local and state levels have led to an increase in face mask use by the general public, which has exacerbated demand and heightened the global supply shortage of viral-filtering facemasks [1]. For example, in South Korea an increase in PPE supplies by 40% per month from the current compounded annual growth rate (CAGR) of 6.5% is projected and not expected to decline post-pandemic, but rather to increase to a 20% CAGR by 2025 [5]. In particular, the scarcity of N95 facemasks [6] has forced healthcare workers to endure and adopt measures beneath the standards of the United States Center for Disease Control (CDC) and Food and Drug Administration (FDA) guidelines [7, 8]. In hospitals and facilities across the nation, healthcare workers left with few options opted for makeshift alternatives or repeated usage of disposable facemasks [1]. Unfortunately, many of these alternatives, including the reuse of traditional disposable facemasks, jeopardizes the protective effect and may even increase the risk of infection [1, 6]. With supply constraints and escalated concerns of infection, it has become increasingly clear there is an urgent need for elevated production of PPE, especially reusable facemasks. Disposable masks, when used by the general public, and industries outside of our healthcare systems, contribute to the biomedical waste (BMW) that has built up post COVID known as COVID-waste. Considered a new category of BMW, COVID-waste can act as a vector for SARS-CoV-2, the virus known to infect and cause COVID-19. It has been reported that the virus can survive up to 7 days on things such as facemasks; therefore, proper disposal or sterilization is needed by the general public [5]. It has been estimated that within the United States alone, an average years’ worth of waste could be generated in just 2 m if reusability, recycling and/or policymaking measures are not put into place [9] The continued increase in the number of people infected with COVID-19, within different regions and countries around the globe, indicates the world will be overrun by COVID-waste, ultimately having a deep impact on sustainable waste management practices in the near future [5].

In order to combat the shortage of disposable PPE, its contribution to COVID-waste, as well as the increased demand of facemasks and other types of PPE in general, the 3D printing industry has rapidly developed, modified, and disseminated 3D printed personal protective equipment [6, 10–14]. The production of PPE using 3D printing technology has allowed for effective, inexpensive, and reusable products to be rapidly produced and deployed to industries as well as the general public. 3D printing has significant advantages over traditional manufacturing, as it offers more expedience, economic value and variable production processes. 3D printed PPE can provide healthcare workers and the general public with inexpensive, scalable products capable of protection against infectious matter [14]. Of particular concern, however, is the sterility of 3D printed materials, as well as the ability to re-sterilize and safely reuse 3D printed PPE. Based on the standard Infection Prevention and Control (IPC) guidelines, PPE is a disposable, single-use item. While there are no proven effective methods of decontamination and reprocessing of traditional PPE [15], there have been reports of intrinsic sterility of 3D printed products due to the extrusion temperatures typically seen in 3D printers being significantly higher than most autoclave cycles [16]. This would suggest upon completion, the newly printed 3D mask is sterile, however the finish of fused filament fabrication (FFF), the most common type of 3D printing, is naturally rough which makes its surfaces more difficult to clean with wipe-down methods.

We look to investigate this intrinsic sterility, as well as the ability to re-sterilize various types of filamentous plastic used to make 3D printed PPE. The most prevalent designs for 3D printed PPE feature an internal lattice structure often made of PLA or TPU, referred to as an “infill”. While this feature aids in the stability and durability of the final product, it is the authors opinion that re-sterilization of these products for repeated use is difficult due to the inherent porosity. In this paper, we describe the efficacy of using a novel, single-walled, polypropylene based approach to 3D printed PPE. Polypropylene is not known to be a commonly used material for 3D printing and currently none of the PPE being made for the COVID-19 pandemic has referenced using it [14]. Furthermore, the novel, single-walled design could serve as a replacement model for the more prevalent “infill” design commonly used. We feel that, due to a simpler internal structure, single-walled polypropylene objects may be capable of being fully sterilized following their production through conventional methods such as autoclaving, or with the use of chemicals such as household bleach, rubbing alcohol and/or hydrogen peroxide (Table 1). Furthermore, production of these objects is likely to be more expedient and inexpensive compared to their multilayered counterparts and offer an alternative solution during a nationwide scarcity of N95 masks in the industrial and biomedical field. The design also allows custom scaling for various size fits and polypropylene is more malleable to the face compared to other 3D masks printed using different plastics.

**Table 1:** General Characteristics of Common 3D-printing Materials for Use in PPE

Material	Maximum Temp*	Basic Characteristics	Biocompatibility	Chemical Cleaning Concerns
Polypropylene	82.2°C Higher temps may compromise material	Relatively cheap thermoplastic. Can be used as both a plastic and a fiber	Currently used for: suture material, meshes, laboratory containers, drug delivery systems	Good chemical resistance (www.hmcpolymers.com), highly resistant to alcohol and solvents, negligible effects in bleach.
PLA	60-65°C Not suggested to heat sanitize	Good all-purpose FFF printing filament	Additives may not be biocompatible	Alcohol and solvents will degrade item
TPE/TPU	Do not apply heat	More flexibility	Additives may not be biocompatible	Alcohol and solvents will degrade item

*General guidelines, properties may vary slightly among brands

Table modified from: Covid-19|Healthcare Coalition (https://c19hcc.org)

In addition, we investigate the ability for complete re-sterilization without the use of an autoclave in PPE created utilizing the polypropylene, single walled design, vs. those created with the PLA or TPU infill lattice structure. Since 3D printers are being utilized by average households’ the authors wanted to test re-sterilization using everyday household cleaning products. It is worth noting that as a plastic polypropylene has been thoroughly tested for chemical resistance using over 260 chemicals and substances, including the ones analyzed here, and are available to the general public [17]. It is advised that consultation with hospital guidelines on the frequency, nature and acceptableness following disinfection or sterilization of reusable equipment take place before use. Information regarding the use of 3D printed PPE and medical supplies made from common 3D printing materials can be found here: https://c19hcc.org/. We hope this data will help broaden future efforts in 3D printing and help narrow focus on the use of single walled polypropylene designs. We also hope to fill a gap in the literature regarding the inability to fully re-sterilize 3D printed medical supplies, such as PPE that uses the infill-based design, by means of introducing a better printing material, polypropylene. In addition, by proposing a more optimal, single-walled, design for 3D printed PPE, we are thus offering a new “reusable 3D printed PPE platform” for the general public, industries and the healthcare field alike. Finally, we hope that re-usable PPE. such as this, offers an improved environmental alternative through a reduction in the amount of biomedical waste (BMW), specifically COVID-waste, being generated.

## Methods

### 3D printing techniques and materials

Fused filament fabrication (FFF), an accessible 3D printing method was used. Polymer filament was extruded through a heated nozzle (0.4mm nozzle bore) onto a build platform to build the samples layer by layer. The material used as feedstock or the FFF polymer were all 100% dense, 1.75mm diameter, 1kg spools of the following: polypropylene filament, item number SMPPL0NT0A075, from “Smart Materials” in Spain (https://www.smartmaterials3d.com/en/pp-filament), PLA was Push Plastic PLA from https://www.pushplastic.com/products/pla-1kg and TPU was Push Plastic Flex TPU 95a from https://www.pushplastic.com/products/flexible-tpu?variant=27607340807. The bed adhesive used was from the same company and is their “Smart Stick” adhesive for polypropylene, item number PVP2000. The 3D printer model used was the Original Prusa MK2S 3D printer from Prusa Research based in Prague, Czech Republic. The program used to generate machine commands, or “Gcode” from the 3D model was “Simplify 3d”, a windows program that takes a 3D model and converts it into code for the 3D printer (https://www.simplify3d.com). 3D printing was accomplished using the “vase mode” for single wall design, which required no support structure. Steps for recreating the 3D Sani-Mask process can be found in supplemental material.

### Initial testing of PLA sterility and sanitizing

**Fig. 1:**
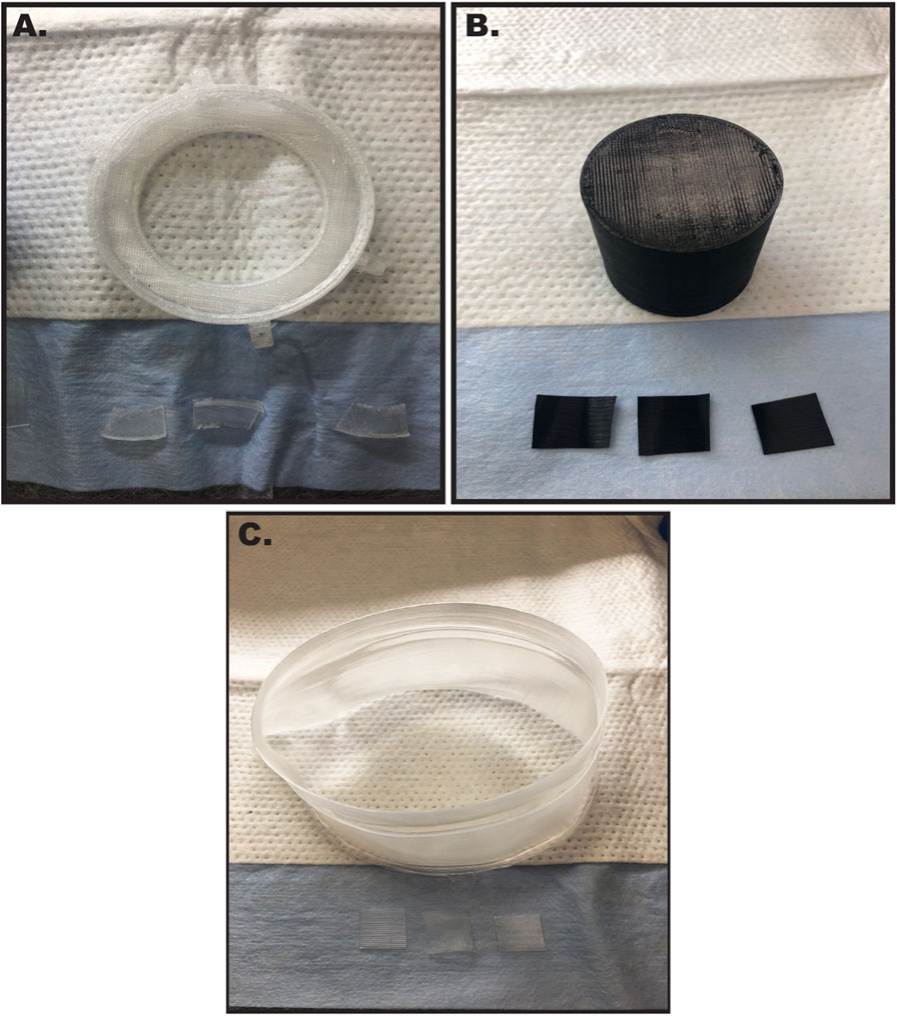
Representative Images of PLA, TPU, and polypropylene samples. 3D printed infill-based cylinders (4cm length and 4cm diameter) and 1.25cm single wall design 3D printed squares utilized in these experiments composed of **A**) PLA or **B**) TPU, and **C**) polypropylene

3D printed cylinders (4cm length with 4cm diameter) were printed with infill as described above (Fig. 1a-c). These cylinders were then incubated, or sanitized for 20-min with 200 mL isopropanol (91%) then placed on sterile gauze and allowed to dry. This was followed by an incubation in 1L sterilized beakers with 125 mL sterile Lauria-Bertani (LB) broth at 37°C for 24- and 72-h. Following incubation, bacterial growth was determined by measuring the optical density (OD_600_). Gram staining was preformed to determine the presence of gram-positive or –negative bacteria.

### PLA, TPU and polypropylene same day sanitizing

One point twenty-five centimeters squares of PLA, TPU, and polypropylene were printed using the single wall design techniques listed above (Fig. 1a-c). These squares were then initially inoculated with 10^7 *cfu* concentrated competent DH10b *E. coli* in liquid broth overnight, and then subjected to various sanitizing methods. Control squares were not sanitized. The first sanitizing method was with 3% bleach for 20 min, followed by 3% hydrogen peroxide for 20 min, and then rinsed in ddH_2_O for 20 min. The second sanitization method was with 3% bleach for 20 min followed by a rinse in double distilled H2O (ddH_2_O) for 20 min. The third sanitizing method was with 91% isopropanol for 20 min followed by a rinse with ddH_2_O for 20 min. All samples were then placed on sterile gauze to dry. The squares were then incubated in 150 mL sterile Erlenmeyer flasks with 15 mL of sterile LB broth for 24 and 72 h. Bacterial growth was then determined by measuring OD_600_. LB broth incubated without squares was used as a control for inherent broth sterility.

### Polypropylene same day, 24, and 48-h sanitizing

One point twenty-five centimeters squares of polypropylene were printed using the single wall design techniques listed above. These squares were then inoculated with 10^7^ *cfu* concentrated competent DH10b *E. coli* in liquid broth overnight*.* For the same day sanitizing, the squares were sanitized with 3% bleach for 20 min and rinsed with sterile ddH_2_O for 20 min. All samples were then placed on sterile gauze to dry.

For 24 h sanitizing, squares were inoculated with 10^7^ *cfu* concentrated competent DH10b *E. coli* in liquid broth overnight. One set of triplicate samples were sanitized with benzalkonium chloride for 5 min, followed by incubation at 70°C for 24 h. A second set of samples were sanitized with 3% hydrogen peroxide (H_2_O_2_) for 20 min and incubated at 70°C for 24 h. A third set of samples were sanitized with 3% H_2_O_2_ for 20 min and incubated at 70°C for 24 h.

For 48 h sanitizing squares were inoculated with 10^7^ *cfu* concentrated competent DH10b *E. coli* in liquid broth overnight. One set of triplicate samples were sanitized with dish soap in ddH_2_O for 20 min and then incubated at 70°C for 48 h. A second set were sanitized with benzalkonium chloride for 5 min, followed by incubation at 70°C for 48 h. A third set of samples were sanitized by rinsing with ddH_2_O for 20 min followed by incubation at 70°C for 48 h.

Following the sanitizing methods above, the polypropylene samples were incubated in sterile 10mL glass test tubes with 3 mL sterile LB broth at 37°C for 24- and 72 h. Bacterial growth was determined by measuring OD_600._ Autoclaved squares and LB broth without squares were used as controls.

### Gram staining

Glass microscope viewing slides were labeled with circles using a wax pen. An inoculating loop was flamed to sterilize. The cooled loop was dipped into LB broth with bacterial growth. Briefly, bacterial culture drops were placed inside the wax circle on labeled glass slide. The samples were allowed to dry for 20 min. Heat fixation was accomplished by passing the slide over a Bunsen burner flame. The heat fixed slides were stained with a gram stain kit per manufacturer’s instructions (Remel, Lenexa, KS). Following the Gram Stain procedure, slides were air dried and gram stained bacteria was visualized using a light microscope.

### Statistical analysis

Statistical analysis was conducted using GraphPad Prism v8.2.1 (GraphPad Software INC.). For comparisons with two groups, data was analyzed with multiple t-tests, and discovery was determined using the two-stage linear step-up procedure of Benjamini, Krieger and Yekutieli, with Q = 1%. For comparisons with 3 or more groups, data was analyzed using 2way ANOVA and Tukey’s multiple comparisons test. A *p*-value of less than 0.05 was considered significant.

## Results

### PLA cylinders sanitized using a 20-minute isopropanol sanitization protocol showed robust bacterial growth

**Fig. 2:**
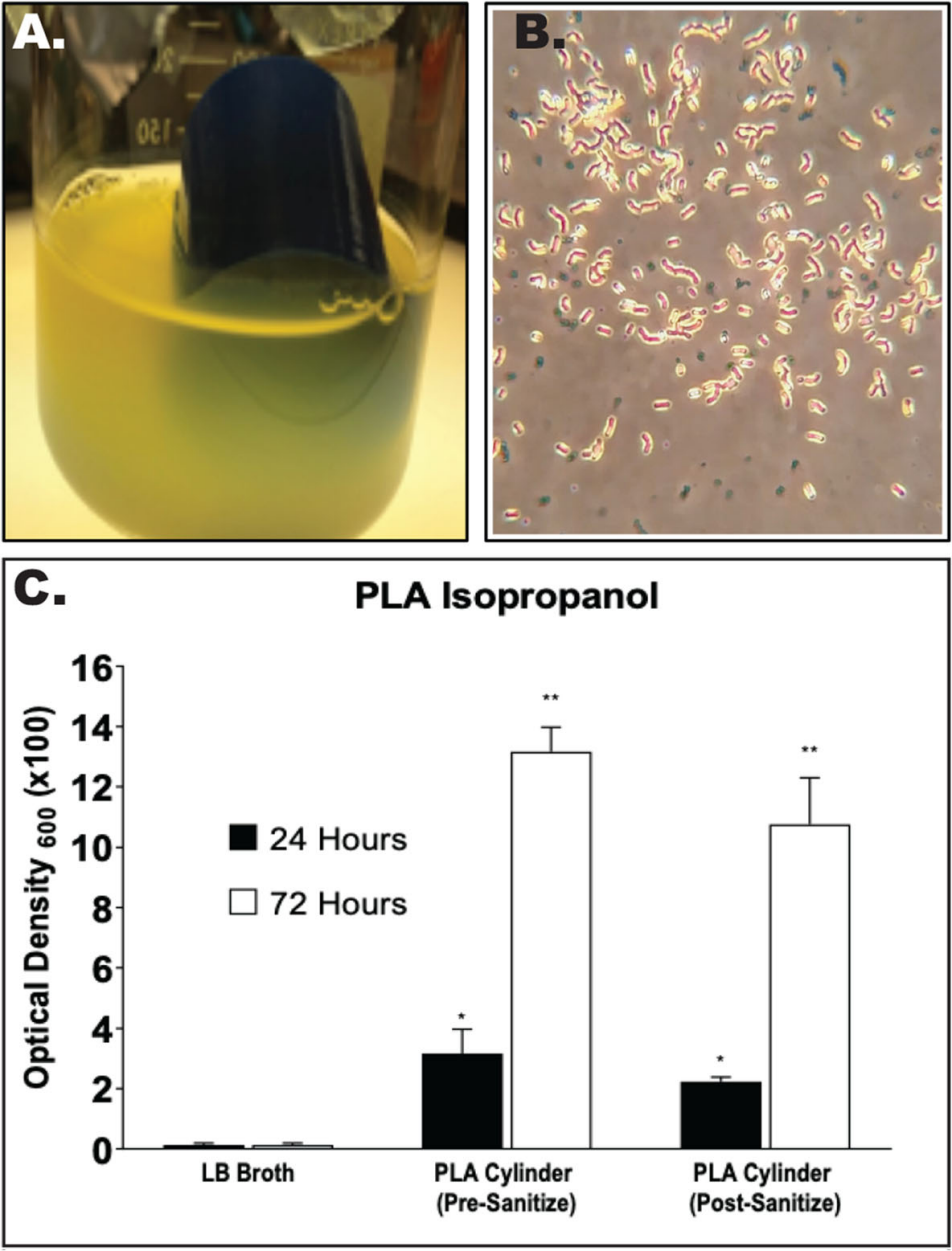
3-Dimensional printing using Polylactic Acid and infill design allows for bacterial growth within 24h of sterilization. PLA-based 3D-printed cylinders with infill were incubated for 24 and 72 h in sterile 1-Lieter beakers and 125mL sterile LB broth following sanitization using 200mL isopropanol (91%) for 20 min. **A**) Representative image of 24 h post sanitization, showing robust bacterial growth. **B**) Gram staining revealed both gram-positive cocci and gram-negative rods. **C**) OD600 was determined to quantify the relative bacterial growth pre- and post-sanitization. Significant bacterial growth was observed at both 24-h and 72-h timepoint. * *p* < 0.05; ** *p* < 0.01 when compared to LB broth control

Following the printing procedures outlined in methods, PLA cylinders (Fig. 1a-c) printed with infill were sterilized using 91% isopropanol, followed by incubation at 37°C in sterile LB broth for 24 and 72 h. At both 24 and 72 h, robust bacterial growth was observed within the liquid broth (Fig. 2A). Gram staining of samples from the broth revealed the presence of both gram-positive cocci as well as gram-negative rods (Fig. 2B). Quantification of growth revealed significant increases (*P* < 0.05) in bacteria at both 24 (OD_600_ = 2.2) and 72 h (OD_600_ = 10.8) when compared to LB broth alone (Fig. 2C).

### Sanitizing of 3D printed materials following bacterial inoculation is possible for single-walled polypropylene, but not for PLA or TPU based products

**Fig. 3:**
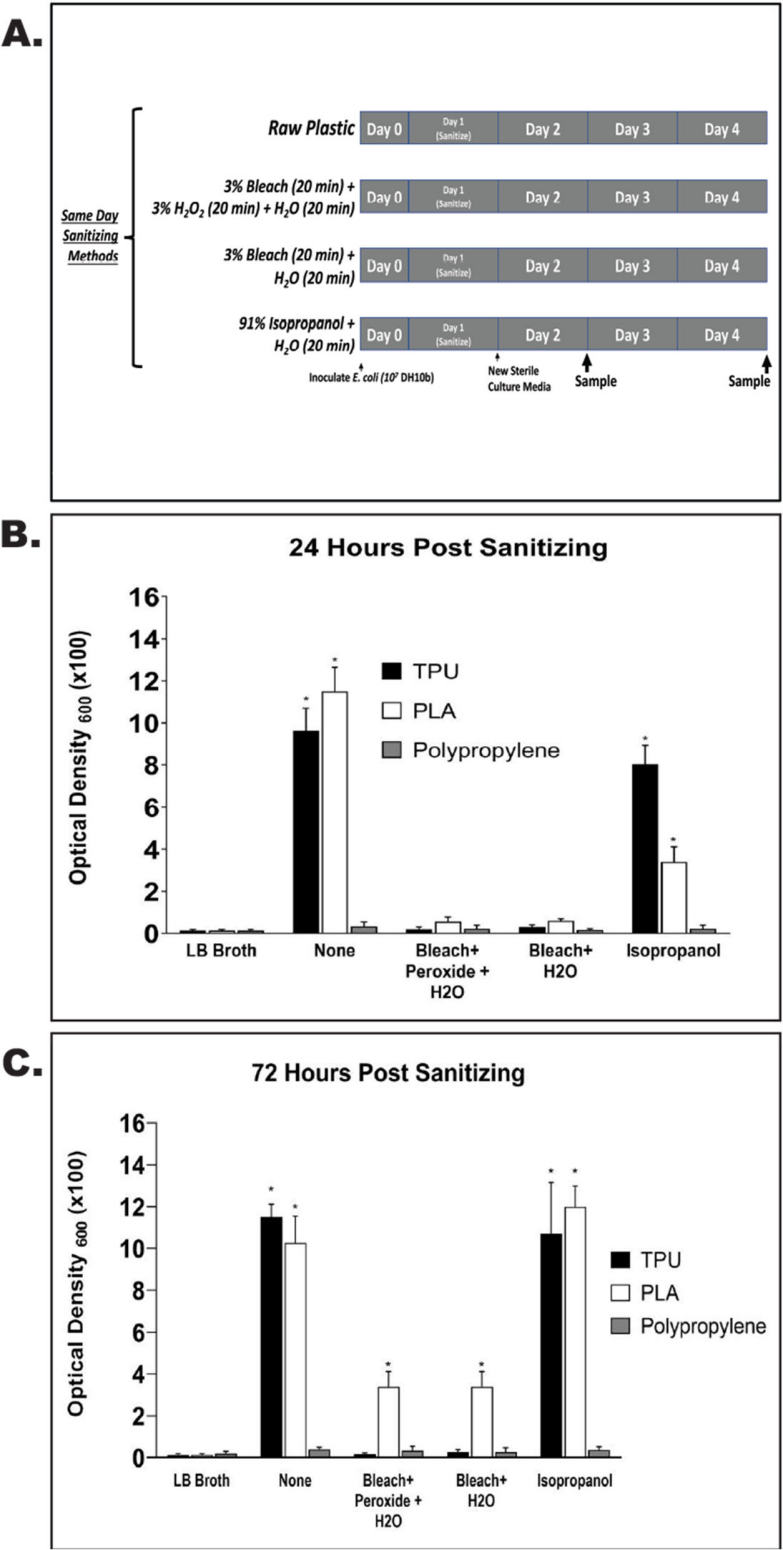
Comparison of TPU, PLA, and Polypropylene Sanitization. **A**) Schematic representation of experimental design to include timing and method of sanitization protocols. **B**) Quantified bacterial growth from each plastic type following 24 h of incubation in LB broth at 370C. * indicates a *p* < 0.5 when compared to LB broth control. **C**) Quantified bacterial growth from each plastic type following 72 h of incubation in LB broth at 370C. * indicates a *p* < 0.5

In order to determine if polypropylene is a better alternative plastic for the production of 3D printed PPE, 1.25 cm square samples of single wall design 3D printed PLA, TPU, and polypropylene were inoculated overnight with *E. coli*, chemically sanitized, and then incubated in liquid broth for 24 and 72 h (Fig. 3a-c). At both timepoints, there was robust bacterial growth for both the PLA and TPU un-sanitized samples (PLA OD_600_ = 11.5 at 24h and 10.3 at 72h; TPU OD_600_ = 9.6 at 24h and 10.3 at 72h), in comparison, single-walled polypropylene showed a significant inhibition of growth (OD_600_ = 0.3 and 0.4 at 24h and 72h), respectively. Isopropanol sanitization did not prevent bacterial growth for either PLA or TPU based samples (OD_600_ = 3.4 and 8.0 at 24h and 12.0 and 10.7 at 72h, respectively), while polypropylene samples had very little growth at 24 or 72 h post-sanitizing (OD_600_ = 0.2 and 0.4), respectively. Additionally, while bleach alone, and bleach plus peroxide were able to inhibit bacterial growth at the 24 h timepoint in all sample types, significant growth was observed in PLA based samples at 72 h (OD_600_ = 3.4 for both treatments).

### Re-sterilization of single-walled polypropylene is possible using household disinfecting and cleaning solutions and methods

**Fig. 4:**
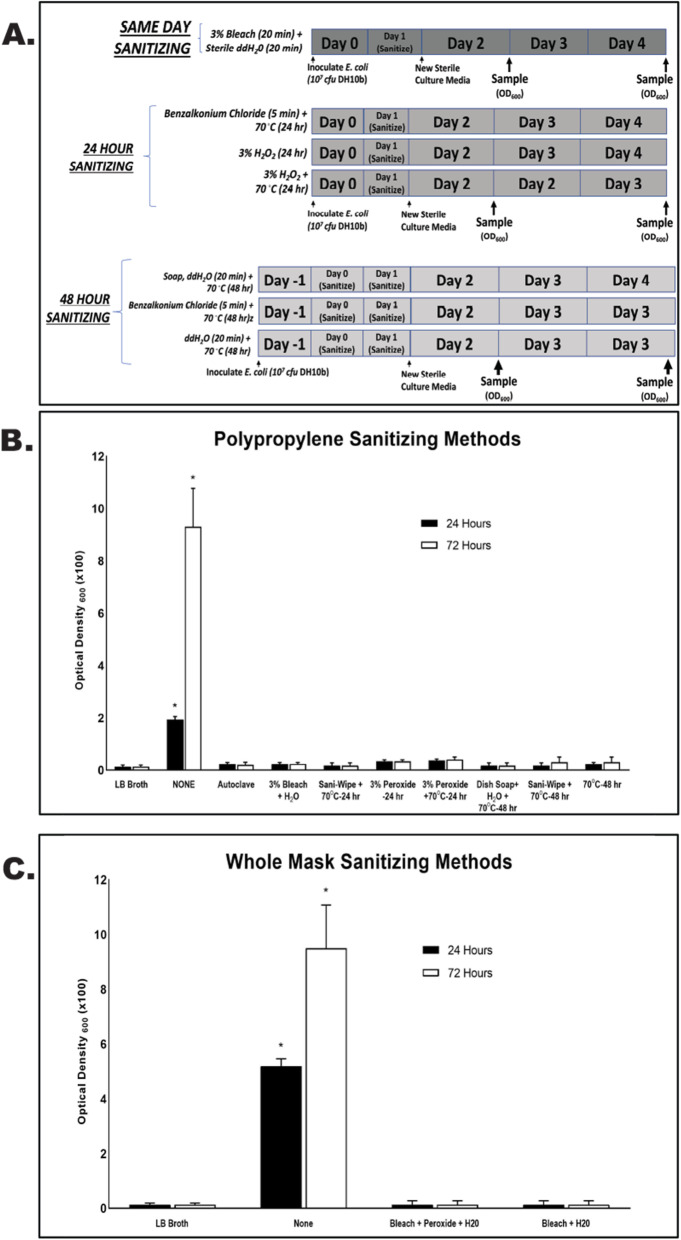
Sanitization of Polypropylene based samples and whole masks. **A**) Schematic representation of experimental design to include timing and method of sanitization protocols. **B**) Quantified bacterial growth from each sterilization method type following 24 and 72 h of incubation in LB broth at 370C. * indicates a *p* < 0.5 when compared to LB broth control. **C**) Quantified bacterial growth from polypropylene based whole masks following 24 and 72 h of incubation in LB broth at 370C. * indicates a *p* < 0.5 when compared to LB broth control

To determine if the general public can sanitize 3D-printed single-walled polypropylene, 1.25cm squares, as well as entirely polypropylene based 3D printed masks were inoculated with *E. coli* for 24 h. Following inoculation, they were sanitized with various household solutions and methods, and placed back into sterile LB medium and incubated at 37°C for 24 or 72 h. Furthermore, we tested the ability to sanitize the single walled polypropylene samples with autoclaving, as polypropylene has been shown to withstand typical autoclave cycle temperatures and pressures, while PLA and TPU cannot. Regardless of the re-sanitizing solution or method used, including autoclaving, no significant bacterial growth (*p* < 0.05) occurred except in controls (no-sanitizing methods (*p* > 0.05) when compared to LB broth alone (Fig. 4).

## Discussion

The emergence of the COVID-19 pandemic has led to an increased demand for personal protective equipment for not only healthcare workers, but also for the general public. As airborne respiratory particles are thought to be the biggest threat for viral transmission, the use of facemasks has become increasingly important, and created an unmet demand for affordable and reliable masks. The 3D-printing community has stepped up and created PPE for distribution to both the health care fields, as well as the general public. However, it was unclear whether these masks were sanitary, and if they could be re-sterilized for multiple uses.

Our study shows that while PPE produced using PLA and the traditional infill-based patterns model may be initially sterile, re-sterilization is not possible using methods such as isopropanol, bleach, and/or H2O2. This is likely due to the inability of the crypts and small spaces created by the infill to be thoroughly reached by cleaning solutions. In as short as 24 h after sanitizing, measurable bacterial growth can be observed in samples created using PLA and TPU plastics. In addition, autoclaving is technique typically utilized to sanitize a variety of materials, most in the general public do not have access to an autoclave, and the use of heat is not recommended for PLA and TPU 3D printed items (Table 1). Polypropylene, however, has been used in clinical and laboratory settings, and is commonly autoclaved. Thus, the combination of polypropylene and a single-wall design show a much greater ability to be thoroughly sanitized using either commercial or more general cleaning solutions, with even commercially available dish soap preventing bacterial growth. The elimination of the infill limits the porosity of the material and creates a single surface for bacteria to reside. These styles of PPE can therefore be thoroughly re-sanitized at home for personal use, or by using autoclave based sterilization in a hospital setting, providing a distinct advantage for polypropylene over TPU and PLA.

In regard to safety, polypropylene has been FDA-approved for food contact, has a high heat tolerance, is BPA free, and has even been used in some surgical devices and implants. A recent study investigated the baseline toxicity, oxidative stress response, endocrine activity, and cytotoxicity of polypropylene, which performed well in all their studies [18]. While not FDA approved, the FDA has issued guidance on the technical considerations for additive manufactured medical devices, including 3D-printed materials [19]. In addition, the FDA has released additional guidance on the production of 3D printed medical supplies, including PPE, during the COVID-19 pandemic, which should be considered when designing or printing any medical devices or PPE [20].

The ability to create inexpensive, re-usable, and sterilizable PPE using 3D printing techniques is essential not only for our ability to slow the spread of infection during the COVID-19 pandemic, but also to reduce the amount of COVID-waste generated by disposable facemasks. Due to the nature of 3D printing, there is great variability in sizing. With our model, the 3D engineer is capable of scaling to precise measurements helping to accommodate individual needs for a more custom fit. Furthermore, the filter portion of the single-walled polypropylene face shield is removable and replaceable; thus, it provides the wearer with limitless options when selecting filtering power. This provides the public the opportunity to use common household items such as simple folded paper towels, shop towels and cotton rags. It also offers healthcare professionals the opportunity to upgrade to medical grade filters as directed. When contaminated the filter is simply removed and replaced, allowing the wearer to better protect themselves and contribute less COVID-waste compared to disposable masks. By providing a product that is quick to make, cost effective, versatile, and reusable we can help protect the general public, health professionals on the front lines as well as the environment. 3D printing offers communities one of the most cost- and time-effective ways to meet the increased demand of PPE. Demonstrating that masks and other PPE can be created with efficacy, and have the ability to be sterilized after use, could lead to the production of powered air purifying respirators (PAPRs) from 3D printers, thus revolutionizing the surgical and intensive care PPE worn by professionals. Hospitals might begin to use 3D printers to manufacture their own supplies rather than rely on outside sources and government allocations, which can have enormous economic implications. The ease of sterilizing the masks would allow effective use by the general population and industries as well, leaving the more traditional products for the healthcare workers. Ultimately 3D printing using sterilizable single walled materials such as polypropylene, allows for a quick response in the case of mask shortages in healthcare setting and general populations. As we move into the flu season, and with COVID-19 infection rates being at their highest points yet, we must look to better alternatives, such as 3D printed PPE that will reduce burden on the already stressed manufacturing and PPE supply chains. Having such alternatives will only improve people’s chances in the case of another pandemic or the continued resurgence of the current one we are still in. It is also a better alternative for our environment and would contribute less to COVID-waste and the pressure forecasted to be on our sustainable management systems in the future.

## Conclusions

The use of polypropylene in a single wall design for 3D printing allows for the creation of inexpensive and rapidly produced PPE for both healthcare workers and the general public. This is a better alternative than PLA and TPU based 3D printed products due to the inability to thoroughly re-sterilize them following contamination. Here we show that 3D printed PPE produced with a single wall design can be sanitized using a variety of chemical solutions, including household cleaners, as well as via autoclave, providing a distinct advantage in health care settings over PLA and TPU based designs. 3D printed polypropylene masks are also a better alternative for the environment, due to their ability to be re-sterilized thus reducing the waste of single use masks used by the general public and within the healthcare industry. It is worth mentioning that repeated exposure to submergence in a chemical disinfectant was not tested in this research. The COVID-19 Healthcare Coalition published concern regarding disinfection techniques by submersing 3D printed items. Specifically, that repeated submersion may weaken 3D printed items and could increase their porosity [21]. Since our design is printed using polypropylene, an untested 3D printing material, and a single walled design, eliminating the infill and porosity, submergence testing and weakening of the mask material still need to be tested.

## Supplementary Information

**Additional file 1:.** 3D Printing Single Walled Polypropylene Parts for Creating Sterilizable Objects

## Data Availability

All data generated or analyzed during this study are included in this published article.

## Abbreviations

3D: Three-dimensional
BMW: Biomedical waste
CAGR: Compound annual growth rate
CDC: Center for Disease Control and Prevention
COVID: Coronavirus
COVID-19: Coronavirus disease of 2019
ddH2O: Double distilled water; highly pure water
FDA: Food and Drug Administration
FFF: Fused filament fabrication
H2O2: Hydrogen peroxide
LB: Lysogeny broth
OD: Optical density
PAPRs: Powered air purifying respirators
PPE: Personal protective equipment
PLA: Polylactic acid
SARS-CoV 2: Severe acute respiratory syndrome coronavirus 2
TPU: Thermoplastic polyurethane
