# Supplementary material for: Sterilization and sanitizing of 3D-printed personal protective equipment using polypropylene and a Single Wall design
Source: 3D Print Med. 2021 Jun 11;7:16. doi: 10.1186/s41205-021-00106-8 (PMC8193021; doi:10.1186/s41205-021-00106-8)
Supplement: Supplementary file 1 — Additional file 1:. 3D Printing Single Walled Polypropylene Parts for Creating Sterilizable Objects [file 41205_2021_106_MOESM1_ESM.pdf]

3D Printing Single Walled Polypropylene Parts for Creating Sterilizable Objects

This guide endeavors to thoroughly outline the steps used to create sterilizable objects from single-walled 3d printed polypropylene.

The material used as feedstock was a 1kg spool of 1.75mm diameter polypropylene filament from "Smart Materials" in Spain. The bed adhesive used was from the same company, and is their "Smart Stick" adhesive for polypropylene.

The 3d printer model used was the Original Prusa MK2S 3D printer from Prusa Research based in Prague, Czech Republic.

The program used to generate machine commands, or "Gcode" from the 3d model was "Simplify 3d".

Steps to recreate process

1. Download and install Simplify3d

2. Once the program is installed, follow the tutorial at https://help.prusa3d.com/en/guide/how-to-import-profiles-to-simplify3d-4-x-windows-macos_18360 to import the needed settings and profiles for the MK2S 3d printer

3. In Simplify3d click the "import" button

4. Navigate to the location that the STL 3d model is located on your computer, and open the file

5. Click "Add" at the bottom left of the screen to add a profile to use for the print

6. Select the Original Prusa i3 MK2S from the "select profile" dropdown menu

7. Select "PLA" from the "Auto Configure for Material" dropdown menu

8. Click the "Show Advanced" button at the bottom left of this screen

9. Navigate to the "Layer" tab

10a. Click the checkmark box near the bottom of this tab named "Single outline corkscrew printing mode (vase mode)". Ensure this box is checked

10b. Change the "Top Solid Layers" and "Bottom Solid Layers" setting to 0

10c . Navigate to the "Cooling" tab.

10d. Select layer 2 from the window on the right and click "Remove Setpoint". This ensures the part cooling fan does not run during the print

11. Navigate to the "Temperature" tab

12. Click "Primary Extruder" in the Temperature Controller List

13. Select the setpoints under "Per-Layer Temperature Setpoints" One at a time and click "Remove Setpoint" until the box is empty

14. Under "Temperature" input 250 into the box and click "Add Setpoint". This tells the software what temperature to set your printing nozzle to.

15. Navigate to the "Extruder" tab

16. Change the "Extrusion Multiplier" setting to 2.00. This makes the extruder push out more material

17. Navigate to the "Advanced" Tab. Click the check box labeled "Stop Printing at Height". Input 2.00 into the input field. This will make this profile stop printing at 2mm height

18. Click the "Save as New" button near the top of the screen.

19. Save this profile as "Polypropylene1"

20. Navigate to the "Extruder" Tab

21. Set the Extrusion Multiplier to 1.00

22. Navigate to the "Advanced" Tab

23. Uncheck the "Stop printing at Height" box

24. Click the "Start Printing at Height" box

25. Input 2.00 into this box. This will make this profile begin printing at 2mm height

26. Click "Save as New" and save this profile as "Polypropylene2"

27. Close this window

28. Under the "Processes" box in simplify 3d, select each process and click "delete" until the list is empty

29. Click "Add"

30. Select "Polypropylene1" from the "Select Profile" drop down menu and click OK

31. Click "Add" again

32. Select "Polypropylene2" from the "Select Profile" drop down menu and click OK

33. A window named "Select Processes for Printing" will show.

34. Click "Select All" in this window, then OK

35. The software will now beging generating machine commands and preparing them for exporting into a file

36. A new window will be shown that shows a visualization of the toolpath for the part

37. Insert an SD card formatted to FAT32 into your computer

38. Click "Save toolpaths to Disk"

39. Navigate to the SD card and Save

40. Remove the SD card from the computer

41. Plug in the 3d printer and power it on

42. Insert the SD card into the 3d printer's SD card slot located on the left side of the LCD display. Note that the SD card must be inserted with the "back" of the card facing you

43. Click the interface wheel on the printer's lcd and navigate to "preheat"

44. Click "PLA"

45. Wait a couple of minutes for the printer to reach operating temperature

46. Open the spool of polypropylene and install it onto the 3d printer's spool holder located at the top of the machine

47. Using a pair of flush cutters, snip the filament at a 45 degree angle. Snip off the first couple of inches of filament so their are no kinks in it.

48. Click the selection wheel on the LCD and select "Load filament"

49. Insert the filament into the hole at the top of the extruder, applying light downward pressure until the extruder gear "grabs" the filament and begins to pull it down on its own.

50. Material should begin coming out of the hot nozzle.

50b. Remove the long filament string that comes out of the nozzle and discard. Take care not to touch molten plastic or the hot nozzle.

51. The machine will ask you if filament is clear. If material is coming out of the nozzle, select "Yes". If it is not, Select "No". Steps 48-51 may need to be done multiple times until a successful filament load is achieved.

52. Apply a thin layer of "Smart Stick" to the entire printing surface. The printing surface is the large yellowish-clear, rectangular plate.

53. Allow the adhesive to dry for 60 seconds

54. Click the interface wheel and select "Print from SD"

55. Select the file you have prepared and saved

56. The printer will now begin printing the object

57. Once the printer is finished, the object may be pulled from the buildplate by hand.

58. The printer is now ready to receive the next print.
